# An Underwater Robotic Manipulator with Soft Bladders and Compact Depth-Independent Actuation

**DOI:** 10.1089/soro.2019.0087

**Published:** 2020-10-16

**Authors:** Zhong Shen, Hua Zhong, Erchao Xu, Runzhi Zhang, Ki Chun Yip, Lawrence Long Chan, Leo Lai Chan, Jia Pan, Wenping Wang, Zheng Wang

**Affiliations:** ^1^ Department of Mechanical Engineering, The University of Hong Kong, Hong Kong SAR, China.; ^2^ Department of Computer Science, The University of Hong Kong, Hong Kong SAR, China.; ^3^ State Key Laboratory of Marine Pollution and Shenzhen Key Laboratory for the Sustainable Use of Marine Biodiversity, City University of Hong Kong, Hong Kong SAR, China.; ^4^ Department of Mechanical and Energy Engineering, Southern University of Science and Technology, Shenzhen, China.

**Keywords:** underwater robot, soft actuator, underwater manipulation, hydraulic control system

## Abstract

An underwater manipulator is essential for underwater robotic sampling and other service operations. Conventional rigid body underwater manipulators generally required substantial size and weight, leading to hindered general applications. Pioneering soft robotic underwater manipulators have defied this by offering dexterous and lightweight arms and grippers, but still requiring substantial actuation and control components to withstand the water pressure and achieving the desired dynamic performance. In this work, we propose a novel approach to underwater manipulator design and control, exploiting the unique characteristics of soft robots, with a hybrid structure (rigid frame+soft actuator) for improved rigidity and force output, a uniform actuator design allowing one compact hydraulic actuation system to drive all actuators, and a novel fully customizable soft bladder design that improves performances in multiple areas: (1) force output of the actuator is decoupled from the working depth, enabling wide working ranges; (2) all actuators are connected to the main hydraulic line without actuator-specific control loop, resulting in a very compact actuation system especially for high-dexterity cases; (3) dynamic responses were improved significantly compared with the counter system without bladder. A prototype soft manipulator with 4-DOFs, dual bladders, and 15 N payload was developed; the entire system (including actuation, control, and batteries) could be mounted onto a consumer-grade remotely operated vehicle, with depth-independent performances validated by various laboratory and field test results across various climatic and hydrographic conditions. Analytical models and validations of the proposed soft bladder design were also presented as a guideline for other applications.

## Introduction

Underwater sampling is essential for the scientific study of marine life.^1,2^ Conventionally, at least four experienced divers are needed for underwater sampling in the shallow depth water within the photonic zone,^3,4^ especially in tropical waters with prominently vibrant biodiversity.^5^ Considering the high risk and physical limitations for divers in the highly dynamic underwater environment, remotely operated vehicles (ROVs) and autonomous underwater vehicles are actively developed and widely used for underwater exploration and intervention.^6,7^ However, underwater manipulation, with primary focuses in underwater intervention with physical interactions, remains highly challenging, with most commercially available underwater manipulators with either primitive dexterity (1–2 DOFs) or heavy/bulky toward heavy duty applications. The rigidity of conventional rigid body manipulators also makes it very difficult for handling soft and fragile aquatic specimens. Also, high pressure hydraulic actuation and control systems, often heavier and bulkier than the manipulators themselves, are generally required to drive such systems under the water pressure at the working depth.^8–10^ In this case, a substantially large underwater platform is required to provide underwater mobility for such manipulators and the corresponding actuation systems. The escalated size, weight, cost, and serviceability are significantly restricting their wider applications.

Soft robotic offers a new approach to underwater manipulation compared with conventional rigid-bodied robots.^11–15^ In particular, the inherent adaptation and waterproofing of soft actuators are ideal for grabbing delicate and flexible objects underwater,^16–18^ inherited from cable-driven^19^ and biomimetic approaches.^20–23^ Fluidic elastomer actuators also work well in underwater applications, in terms of continuum structures,^24^ hybrid structures,^25^ and even modular structures,^26^ tested in a high pressure environment equivalent to 2300 m depth. Three-dimensional printed soft robotic manipulators have also been proven highly successful in deep-sea operations,^27,28^ tested for more than 2200 m depth,^29^ offering much better compactness and inherent compliance than rigid manipulators for delicate underwater sampling.

However, the state-of-the-art solutions are still compromised between compactness, lightweight, and dexterity, mainly due to the hydraulic actuation and control components for existing soft manipulators are still similar with those of rigid manipulators: (1) for dexterity, multiple actuators require dedicated pressure-regulation feedback loops; (2) for working depth, matching actuation pressure is required to counter the ambient water pressure at the working depth; (3) for fast response time, a high power actuation unit is often needed. Some efforts have been made to address the limitations, from a compact pneumatic system with chemical reactions (not applicable for underwater application)^30^ to jamming grippers with ambient water pressure compensation for object picking with primitive dexterity.^31,32^ In particular, an accumulator was made by Phillips *et al.*^33^ to both counter ambient pressure and increase the flow rate in the actuation level. A spring was used in the accumulator to hold ambient pressure and provide an increased flow rate. Therefore, to achieve compact, lightweight, and fast response simultaneously, innovations are required in the actuation and control systems on the developing stage.

**FIG. 1.**
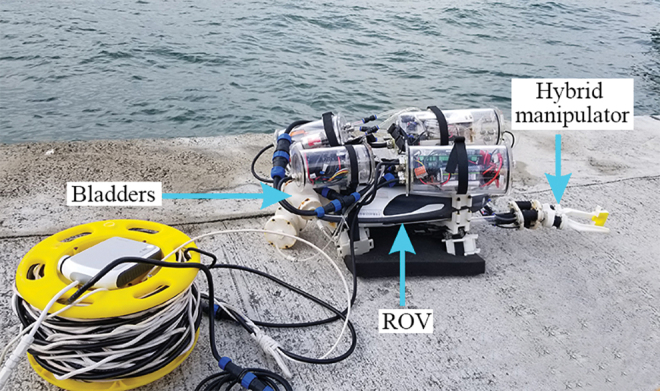
The proposed underwater manipulator with soft bladder-based hydraulic system mounted on a consumer-grade ROV. ROV, remotely operated vehicle. Color images are available online.

In this work, a novel hybrid underwater manipulator framework is proposed, characterized, and demonstrated, with uniform soft actuator design and soft bladder hydraulic actuation (Fig. 1), achieving dexterity, compactness, depth independence, and fast response, by using two compensating soft bladders (CSBs) in the hydraulic actuation system. Complete works on the mechanical design, analytical modeling of the CSB and soft actuators, as well as prototyping work are presented, together with various test results in controlled- and open-water environments in different field sites in the tropical areas with various hydrographic conditions.

The main contributions of this work are the proposed multipurpose CSB concept and the overall soft manipulator actuation and control approach based on it, achieving a unique combination of experimentally verified characteristics distinguishing from the state-of-the-art solutions: (1) high compactness, with CSBs, the entire prototype arm with 15 N payload and 4-DOF dexterity, weighed 2.5 kg (including battery and electronics), and could be mounted onto a recreational ROV platform; (2) depth independence, uniform force output performance across the entire working depth range, irrelevant of ambient water pressure changes; (3) improved dynamic response, nearly twice faster response by using CSBs in the hydraulic loop.

Results on the prototype manipulator are highly promising for a dexterous, compact, and lightweight soft robotic manipulator with nearly uniform payload and dynamic performances across the entire designated working depth range. Following this approach, recreational-grade ROVs could be equipped with easy-to-carry and easy-to-use soft arms, leading to mass applications in both diver assistance and underwater operations.

## Conceptual Design and System Overview

### Design requirements

The targeted design of a soft robotic manipulator for general underwater sampling and manipulation tasks is desirable to be compact, easy-to-operate, with large working depth range, while also being responsive and task-capable. These rather contradicting features are depicted in the following design requirements:

(1) Compactness. The complete self-containing underwater system supporting the manipulator should be able to fit a consumer-grade compact ROV, significantly more compact than existing underwater manipulators. Therefore, conventional hydraulic tanks should be eliminated, and compact pumps/valves are preferred over conventional compressors/cylinders, with minimalistic actuation and control hardware.
(2) Large working depth range. With the limited pressure range from the compact hydraulic components being used, ambient water pressure becomes a vital factor to the hydraulic performance. Special measures are required to ensure the operation and performance of the soft manipulator being independent of the working depth.
(3) Fast response. Fast response and tracking capability are critical for the dynamic tasks of sampling and manipulation. Considering the limited flow rate and response time for the compact actuation components, special measures are required to improve the dynamic response.
(4) Soft arms. Considering the complex underwater environmental factors and the inherent compliant features for soft robots, an easy-to-carry and easy-to-use soft arm is needed for underwater sampling tasks.

### Underwater manipulation system overview

**FIG. 2.**
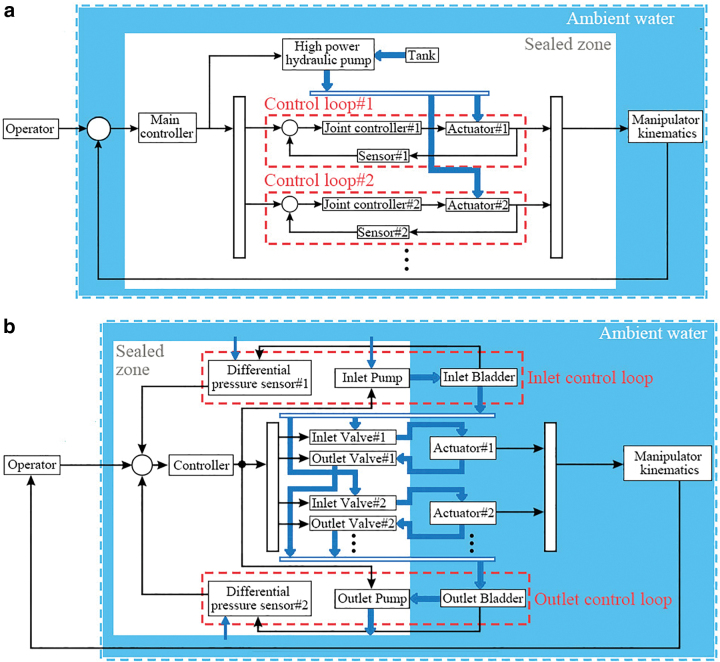
Comparison between the conventional underwater manipulation system and the proposed CSB-based manipulation system. **(a)** The conventional underwater manipulation system. The whole system is isolated from ambient water so that an oil tank is needed as a liquid source. At least one high power hydraulic pump is required to counter ambient water pressure. High power actuation units are also needed for fast response, and each actuator requires an individual feedback loop for dexterous and accurate control. **(b)** The proposed underwater manipulation system. Only electronic components are sealed. A liquid source directly comes from ambient water, and both CSBs and actuators are exposed to ambient water, ensuring that the ambient water pressure is balanced. All the actuators are controlled by the inlet and outlet control loops compactness and simplicity. The improved dynamic response can be achieved by using the elasticity of the two CSBs. CSB, compensating soft bladder. Color images are available online.

The proposed underwater manipulation system was largely modified compared with conventional hydraulic underwater manipulators (Fig. 2) to meet the design requirements. The key features are as follows:

(1) A CSB-based control strategy is used to minimize the use of pumps, valves, and control hardware. Compared with conventional underwater manipulators that use individual control loops for each actuator, the proposed hydraulic system only has two control loops for all the actuators used in the manipulator. As the two CSBs are linked to all the actuators, they act like pressure control units. Also, the CSBs can protect actuators from exceeding the maximum pressure threshold by presetting the working pressure ranges.
(2) The two CSBs are exposed to ambient water to balance ambient water pressure. In this case, the pumps’ working pressure is independent of ambient water depth. Compared with the accumulator used by Phillips *et al.*,^33^ which used a spring to hold ambient pressure, the two CSBs naturally counter ambient pressure, taking advantage of the inherent compliance of soft robots. In this case, the water depth factor can be removed in the developing level.
(3) The inherent flexibility of the two CSBs is used to achieve fast dynamic responses and tracking capability. By setting a working pressure range for the CSBs, pressure differences will be generated between the CSBs and the actuators. In this case, water comes from the pumps can be released with a much larger flow rate. Also, by tuning materials and wall thickness using the analytical model proposed in the Design and Modeling of CSBs and Actuators section, the CSBs can be customized for different applications.
(4) Modularized actuator design is carried out in the underwater manipulation system. As soft actuators’ pressure can be controlled by two CSBs and corresponding valves, sensors are not necessary for each actuator. In this case, only valves and actuators need to be added into the system when calling for more degrees of freedom (DOFs). Also, the modular design helps keep the compactness of the system when DOFs are added.

### The proposed CSB-based hydraulic control framework

**FIG. 3.**
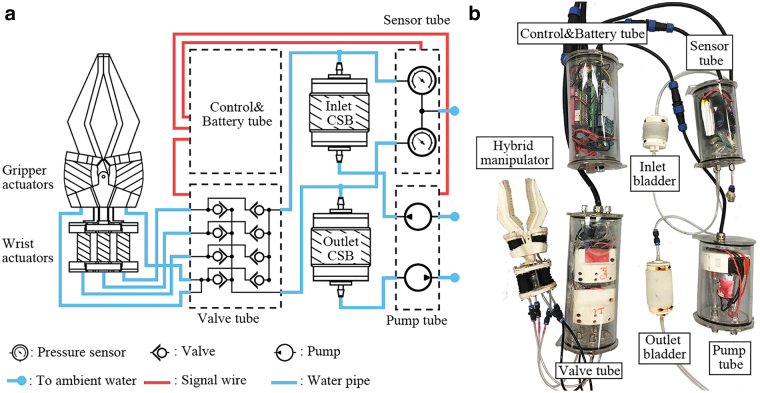
System overview. **(a)** Schematic drawing of the proposed underwater hydraulic system. **(b)** The underwater hydraulic system with the manipulator. Color images are available online.

The proposed hydraulic control framework shown in Figure 3 serves multiple purposes including system compactness, wide working depth range, and dynamic performance. The hydraulic system consists of eight valves (2.5 W each), two pumps (7.2 W each), two CSBs, two pressure sensors, and one control unit. The maximum power consumption is 34.4 W, which is less than half of the power required for standard underwater manipulators.^33^ For the sake of waterproofing and compactness, all the electronic components are put into four acrylic tubes. The four tubes are 100 mm in diameter, and the length ranged from 140 to 250 mm. Overall the whole system, weighted 2.5 kg, can be fit into a 400 × 500 × 250 mm box. The two pumps are connected with two CSBs separately, and ambient water pressure is balanced as the CSBs are exposed to ambient water. Three actuators on the joint correspond to six valves, the rest two valves are connected with the two actuators on the gripper as these two actuators move simultaneously. All the actuators are controlled by two CSBs and the corresponding valves. The system's dynamic performance is improved by presetting two CSBs to pre-established pressure ranges, as the large pressure differences created between actuators and two CSBs will result in a higher flow rate. In this work, the maximum pressure for the CSBs is set to 15 psi due to 3D printed parts limitation.

When the hydraulic system is activated, the two CSBs are pumped to the preset pressure ranges (inlet positive and outlet negative) first. The pressure ranges are determined so that all the actuators can perform within safe working pressure ranges and the response time can be largely reduced. As the two CSBs are exposed to water, they also act like water depth pressure compensators. So whatever the depth is, the two pumps only need to provide pressure difference. When a command signal is received and an actuator needs to be elongated or contracted, the corresponding valves are opened by the control unit and water is released from (inlet)/to (outlet) the two CSBs first. When the pressure in the two CSBs exceeds the preset ranges, the two pumps are activated again to keep the pressure ranges within the preset value.

### Design of the hybrid manipulator

A hybrid design is carried out for the underwater manipulator.^34^ On the one hand, giving complex underwater environments, soft robotics has the advantage of inherent compliance compared with conventional rigid-bodied robots. On the other hand, better accuracy and larger payload can be achieved by applying rigid parts in the design.

**FIG. 4.**
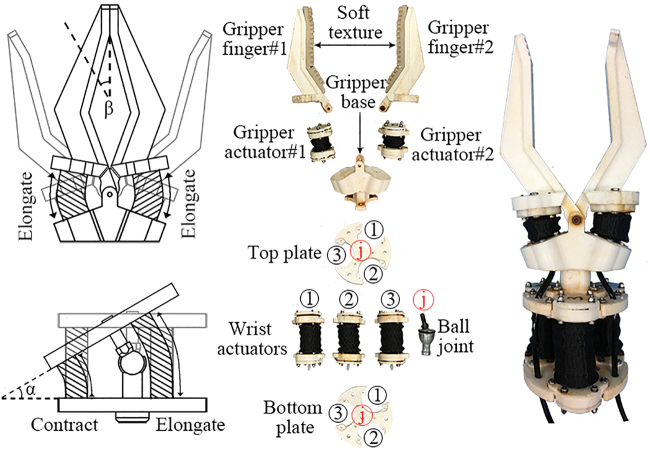
Manipulator characterization. Schematic drawing of the manipulator's motion (*left*). Exposed view of the manipulator (*middle*). The dexterous hybrid underwater manipulator (*right*). Color images are available online.

The underwater manipulator (Fig. 4) consists of two parts: the gripper and the wrist. The gripper has two fingers, both of which are covered with soft textures to increase friction. The two fingers are mounted on the gripper base and two gripper actuators are fixed in between the fingers and the base. The gripper will close when these two actuators are elongated, as these two actuators move simultaneously. The maximum opening angle *β* is 85°. The wrist consists of three wrist actuators, a ball joint, and two 3D printed plates. The top and bottom plates are connected by the ball joint, and three soft actuators are placed triangularly along the edge of the plates. When one actuator is elongated, the other two will be contracted accordingly, which will result in the bending motion along with contracted actuators. The maximum bending angle *α* is 36°.

## Design and Modeling of CSBs and Actuators

As a key component in the hydraulic system, the two CSBs play an unequaled role in the whole system's performance. In this case, the development of the two CSBs is separated from the hydraulic system to elaborate their functionalities in a more detailed manner. The soft actuators, however, share the same importance and similar fabrication process compared with CSBs. In this case, the actuators’ development has been put together with the CSBs for better understanding.

### Design and fabrication of CSBs and actuators

The CSBs proposed in this work serve the purpose of reducing system bulkiness and complexity while optimizing the pump's output so that control response time can be largely reduced. To fulfill these goals, three major requirements need to be satisfied: (1) Ambient water depth pressure can be passively balanced so that the water depth factor can be removed from the developing stage. (2) The pressure differences between the CSBs and the actuators have to be large enough to achieve fast response but not too large to cause damage to the actuators. (3) When pressure is increased, the CSBs need to have enough volume change for the actuators. Taking advantage of the inherent compliance and waterproofing of soft robot, and by exposing the two CSBs in water, ambient pressure no longer needs to be considered on the developing stage. To further investigate the relationship between the performance and the parameters of the CSBs, an analytical model was built up in the Design and Modeling of CSBs and Actuators section. And for the sake of easy investigation, the two CSBs were designed to deform in one axis only (elongation and contraction).

**FIG. 5.**
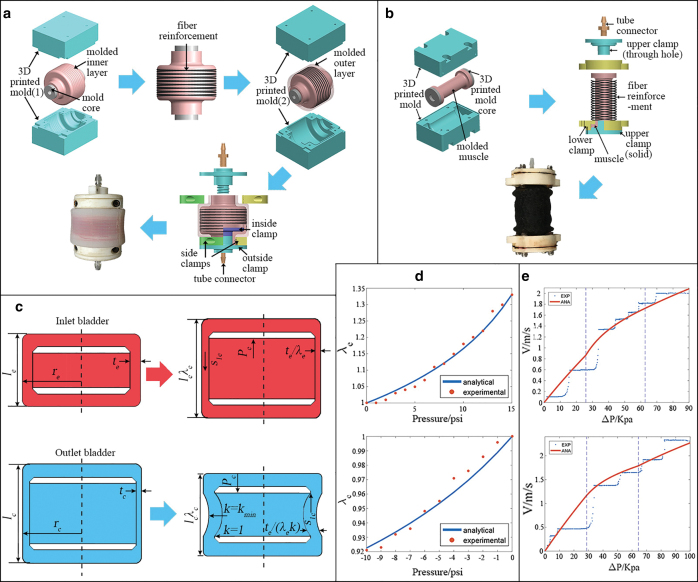
**(a)** CSB molding process (inlet). The outlet CSB molding process is the same except there is no fiber reinforcement. **(b)** Soft actuator molding process. Gripper and wrist actuators follow the same procedure but different lengths. **(c)** Schematic drawing of the two CSBs. The upper one is the inlet CSB (elongation), and the lower one is the outlet CSB (contraction) $\lambda_{e}$ and $\lambda_{c}$ are principle stretch ratios for the inlet and outlet CSBs, respectively. **(d)** CSB pressure versus strain test results (inlet: *upper*, outlet: *lower*). *Blue lines* are analytical results, and *red dots* are experimental results. **(e)** CSB flow speed versus pressure difference test results (inlet: *upper*, outlet: *lower*). ANA, analytical; EXP, experimental. Color images are available online.

There are two CSBs used in the hydraulic system, one in the inlet (elongation) and the other in the outlet (contraction). Both of them share the same shape, but the elongation one has a fiber reinforcement layer, whereas the contraction one is pure silicone. The molding process for the inlet CSB is shown in Figure 5a. The inner layer was first molded (dragon skin 30; Smooth-On, Inc.). After putting on the fiber reinforcement, the outer layer was molded (dragon skin 30; Smooth-On, Inc.). When it was cured, the 3D printed mold core was removed and clamps were added. For the outlet CSB, as there was no fiber reinforcement, it was only molded once (dragon skin 30; Smooth-On, Inc.).

All the five soft actuators used in the gripper and the wrist share the same structure but different length to meet with different requirements, for the purpose of reducing system complexity and realizing modularized actuator design. In particular, the two actuators used in the gripper are shorter to achieve a larger opening angle, whereas the three actuators used in the wrist are longer for larger bending angle. The molding process is as follows (Fig. 5b): The soft chamber was first molded (dragon skin 10; Smooth-On, Inc.) using 3D printed molds. Once the silicone was cured, a fiber reinforcement layer was added to restrict radial expanding. Both of the two sides of the soft chamber were then sealed using 3D printed clamps. Combining the hybrid manipulator design, one can modify the length and inner chamber radius of the actuator based on the demand, without redesigning the whole structure of the soft actuator.

### Analytical modeling of CSBs

To simplify the analytical model, all the materials used in the CSB are considered incompressible, and the Neo-Hookean hyperelastic material model is used. The strain energy density function is given by

(1) $$W=\frac{\mu }{2}\left(I_{1}-3\right),$$

where μ is the initial shear modulus, and *I*_1_ is the first invariant of the three principle stretch ratios $\lambda_{1}$, $\lambda_{2}$, and $\lambda_{3}$:

(2) $$I_{1}=\lambda_{1}^{2}+\lambda_{2}^{2}+\lambda_{3}^{2}.$$

The principal stress is given by

(3) $$s_{i}=\frac{\partial W}{\partial \lambda_{i}}-\frac{p}{\lambda_{i}},$$

where *p* is the Lagrange multiplier. For incompressible materials, we have $\lambda_{1}\lambda_{2}\lambda_{3}=1$. For the inlet CSB (Fig. 5c, upper one), as there is one fiber reinforcement layer, the circumferential strain is considered negligible, which gives $\lambda_{2e}=1$. It is then obtained by

(4) $$\lambda_{1e}=\lambda_{e};\lambda_{2e}=1;\lambda_{3e}=\frac{1}{\lambda_{e}}.$$

For the outlet CSB shown in Figure 5c, as there are two circular 3D printed parts at the two ends, the circumferential strain at those two points is zero, which gives $\lambda_{2c}=1$. While the principal stretch in the circumferential direction at the center of the CSB is *k* (*k* is a constant and 0>k≤1). It can be obtained by

(5) $$\lambda_{1c}=\lambda_{c};\lambda_{2c}=k;\lambda_{3c}=\frac{1}{k\lambda_{c}}.$$

For the sake of simplicity, although the inlet CSB consists of different materials with different initial shear modulus, they are considered as a homogeneous material with effective initial shear modulus $\bar{\mu }$. For the inlet CSB, radial stress is considered balanced through the thickness (i.e., $s_{3}=0$). Considering a similar derivation procedure in the study by Polygerinos *et al.*,^35^ Equation (3) is further simplified to

(6) $$s_{1e}=\bar{\mu }\left(\lambda_{e}-\frac{1}{\lambda_{e}^{3}}\right).$$

For the outlet CSB, substituting Equations (1), (2), and (5) into Equation (3) yields

(7) $$s_{1c}=\bar{\mu }\lambda_{c}-\frac{p}{\lambda_{c}}$$

(8) $$s_{2c}=\bar{\mu }k-\frac{p}{k}$$

(9) $$s_{3c}=\frac{\bar{\mu }}{k\lambda_{c}}-k\lambda_{c}p.$$

For the inlet CSB, the balance is reached at the ends, which gives

(10) $$P_{e}A_{e1}=s_{1e}A_{e2},$$

where *P_e_* is the pressure difference inside and outside the CSB, $A_{e1}$ is the CSBs cross-sectional area, and $A_{e2}$ is the cross-sectional area of the silicone. Assuming the CSB has initial length *l_e_*, radius *r_e_*, and wall thickness *t_e_*, substituting Equation (3) into Equation (10) yields

(11) $$P_{e}r_{e}^{2}=\bar{\mu }\left(\lambda_{e}-\frac{1}{\lambda_{e}^{3}}\right)\left(r_{e}^{2}-{\left(r_{e}-\frac{t_{e}}{\lambda_{e}}\right)}^{2}\right).$$

For outlet CSB, when k=1, the force generated by pressure difference *P_c_* is equal to the force generated by axial stress. When $k=k_{min}$, radial stress is balanced by the pressure difference *P_c_*. It is then obtained by

(12) $$P_{c}A_{c1}=s_{1c}A_{c2}$$

(13) $$P_{c}=s_{3c}.$$

The initial condition of the CSB is given as *l_c_*, *r_c_*, and *t_c_* for length, radius, and wall thickness, respectively. Combining Equations (7) and (9) into Equations (12) and (13) gives

(14) $$\pi P_{c}r_{c}^{2}=\pi \left(\bar{\mu }\lambda_{c}-\frac{p}{\lambda_{c}}\right)\left(r_{c}^{2}-{\left(r_{c}-\frac{t_{c}}{\lambda_{c}}\right)}^{2}\right)$$

(15) $$P_{c}=\frac{\bar{\mu }}{k_{min}\lambda_{c}}-k_{min}\lambda_{c}p.$$

Substituting Equation (15) into Equation (14) gives

(16) $$P_{c}=\frac{\bar{\mu }t_{c}\left(2r_{c}k_{min}^{2}\lambda_{c}^{5}-2r_{c}\lambda_{c}-t_{c}k_{min}^{2}\lambda_{c}^{4}+t_{c}\right)}{k_{min}\lambda_{c}\left(k_{min}r_{c}^{2}\lambda_{c}^{4}-2t_{c}r_{c}\lambda_{c}+t_{c}^{2}\right)}.$$

Assuming pressure in the actuator is *P_a_* and water flows through the CSBs and actuators is incompressible, the Darcy–Weisbach equation is given as

(17) $$\frac{\Delta P}{L}=f_{D}\frac{\rho }{2}\frac{v^{2}}{\bar{D}},$$

where ΔP is the pressure difference between the CSBs and the actuator, *L* is the pipe length, ρ is the water density, *v* is mean flow velocity, and *D* is the pipe inner diameter. For the purpose of simplicity, although there are different pipe fittings and pipes with different diameters, they are considered as one single pipe with an effective length $\bar{L}$. *f_D_* is the Darcy friction factor when it is laminar, $f_{D}=\frac{64}{Re}$, where *Re* is the Reynolds number. When it is turbulence, by applying the Swamee–Jain equation

(18) $$f_{D}=\frac{0.25}{{\left[log\left(\frac{\varepsilon ∕D}{3.7}+\frac{5.74}{Re^{0.9}}\right)\right]}^{2}},$$

where ε is the absolute roughness. Substituting Equation (18) into Equation (17) gives

(19) $$\Delta P=\left\{\begin{array}{cc} \frac{64\rho \bar{L}v^{2}}{2ReD}, & laminar.\\ \frac{0.25}{{\left[log\left(\frac{\varepsilon ∕D}{3.7}+\frac{5.74}{Re^{0.9}}\right)\right]}^{2}}\frac{\rho \bar{L}v^{2}}{2D}, & turbulence. \end{array}\right.$$

## CSB Validation Experiments

The working performance of CSBs is based on two key features: (1) The CSBs need to have large enough deformation to store enough water for the actuators. (2) The pressure difference between the CSBs and the actuators shall be large enough to generate large flow and increase response time. In this case, the CSBs’ pressure versus strain relationship was tested first. Then, the relationship between flow rate and pressure difference was tested with the proposed CSB-based hydraulic system. At last, the whole hydraulic system's performance was evaluated compared with no CSB condition.

### Experimental setup

**FIG. 6.**
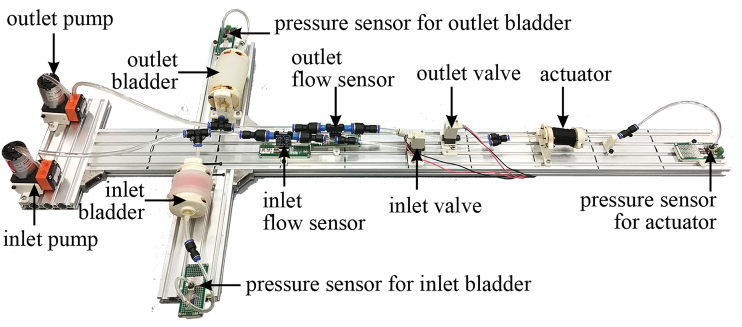
CSB testing platform setup. Color images are available online.

The experimental setup is shown in Figure 6. The hydraulic path in the experiment was the same as the hydraulic system mentioned in the Conceptual Design and System Overview section, except there was only one actuator in the experimental setup. Three pressure sensors (TSCDRRN015PDUCV; Honeywell International Inc.) were used to record pressure for the CSBs and the actuator, respectively. Two flow sensors (FS2012-1001-LQ; Integrated Device Technology, Inc.) were placed in both the inlet path and the outlet path right after the valves for CSBs’ flow rate measurements. The actuator and CSBs used in the experiments were the same as the ones in the hydraulic system and the underwater manipulator. Both the actuator and the two CSBs were fixed to the platform at one end while the other end could freely move. The pipe length that connected the CSBs and actuator to the pressure sensors was set so that when the CSBs or the actuators were deforming, the resistance of bending the pipe was negligible.

### Pressure and strain validation

The pressure versus strain relationship was first tested in this subsection. Principle stretch ratios ($\lambda_{e}$ and $\lambda_{c}$) were measured with every 1 psi pressure increased. This procedure was repeated five times, and the mean value was used for each point. Figure 5d shows the testing results. The two blue curves are analytical models from Equations (11) and (16), and the effective initial shear modulus $\bar{\mu }$ was 65 psi for the inlet CSB and 40 psi for the outlet CSB. Both the models fitted well with experimental data; the root mean square error (RMSE) was 0.012 for inlet CSB and 0.005 for outlet CSB, illustrating that both the analytical models fitted well with experimental data. One can specifically design the CSB with respect to the amount of water needed to be stored by using the pre-established model.

### Flow rate and pressure difference relationship validation

The relationships between pressure difference and flow rate for the two CSBs were tested separately, as the two CSBs had different analytical models. The inlet CSB testing steps were as follows: (1) The pressure for the inlet CSB was set to 13 psi and water began to be pumped into the CSB. The inlet valve remained closed and pressure in the actuator was 0. (2) When the CSB reached the preset pressure, the inlet pump was closed. Then, the inlet valve was opened and water began to flow from the CSB to the actuator due to pressure difference. The flow rate and pressure of the CSB and actuator were measured during this process. (3) When the pressure difference between the CSB and the actuator reached 0, the inlet valve was closed. (4) The above procedures were repeated five times. The outlet CSB followed similar steps except for the outlet CSB was preset to negative pressure and the actuator was filled with water (pressure = 6 psi), so that the pressure difference was also 13 psi at the beginning, and there was enough water inside the actuator for the outlet CSB to suck out. The testing results are shown in Figure 5e; the effective length was 0.5 and 1 m for the inlet CSB and outlet CSB, respectively. Both the analytical and experimental results indicated that for both of the CSBs, with the increase of pressure difference, flow velocity would increase rapidly. However, when the flow became turbulence, the rise of flow velocity significantly reduced when the pressure difference was increased.

### Tracking performance evaluation

Four different system setups were tested to evaluate the system's tracking performance:

S-a: One wrist actuator, one inlet pump, no CSB.
S-b: One wrist actuator, one inlet CSB, one inlet pump.
S-c: One wrist actuator controlled by a hydraulic system with both inlet and outlet CSBs and their corresponding pumps.
S-d: S-c with a shorter gripper actuator.

Three different pressure signals were given to the four setups:

P-a: Was a step function with a 6-s time interval.
P-b: Was a 0.25 Hz sinusoidal signal.
P-c: Was a sinusoidal waveform with increased frequency.

**FIG. 7.**
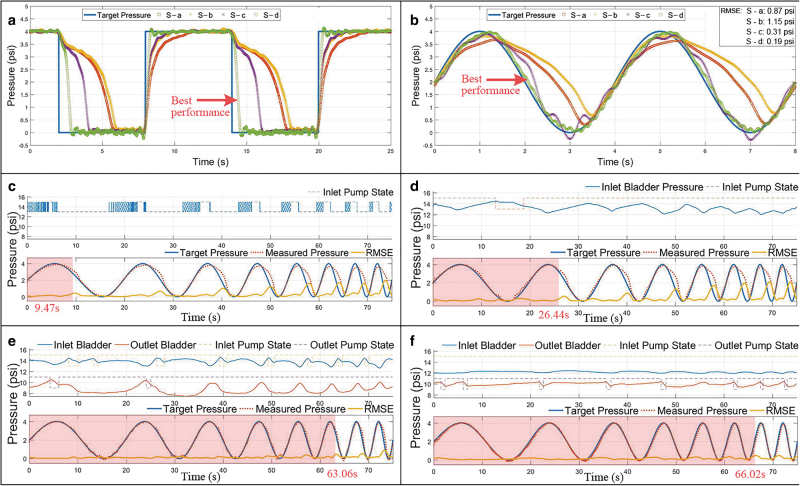
CSB-based hydraulic system tracking performance test results. **(a)** P-a (step function) tracking performance. **(b)** P-b (sinusoidal signal) tracking performance. **(c–f)** P-c (increased frequency with sinusoidal signal) tracking performance for all four system setups. The *red shadow* is the tracking time before tracking failure (critical time). Color images are available online.

The results are shown in Figure 7 and Table 1.

**Table 1.** Tracking Performance Evaluation

System	Rising(s)	Falling(s)	First point of tracking failure(s)
S-a	2.44	3.57	9.47
S-b	1.24	3.85	26.44
S-c	1.25	1.97	63.06
S-d	0.31	0.62	66.02

For P-a tracking (Fig. 7a and Table 1), S-b and S-c halved the rising time compared with S-a, and S-c also halved the falling time compared with S-a and S-b. Apart from that, S-d had a significantly reduced rising and falling time (up to 687%) compared with the rest three systems, illustrating that with the same CSB setup, the smaller the actuator, the faster the charging/discharging rate. In the P-b tracking test (Fig. 7b), S-c and S-d had much smaller RMSE compared with S-a and S-b, and S-d had the smallest RMSE (0.19 psi).

For the sake of comparison and easy understanding, when evaluating the tracking performance of P-c (Fig. 7c–f), we assumed that when the RMSE was below 10% (0.4 psi), the system was able to follow the given signal. Also, the time when RMSE first exceeded, the threshold (critical time) was recorded as a reference. Both S-b and S-c had a larger critical time compared with S-a (Table 1). S-d had similar performance compared with S-c; one possibility could be that although S-d needed less water for the same pressure change, the valve's mechanical properties could not provide enough switching frequency. In summary, it has been proven that the CSB-based hydraulic system can significantly increase system response time compared with no CSB systems.

## Underwater Manipulation System Experiments

According to the design concepts, besides fast response time, the underwater manipulation system also needs to satisfy: (1) compactness and (2) wide working depth range. In this case, the manipulator was fit into a consumer-grade ROV and tested under different depths. Also, a specially designed task was carried out to test the manipulator's dexterity and accuracy.

### Soft actuator experiments

**FIG. 8.**
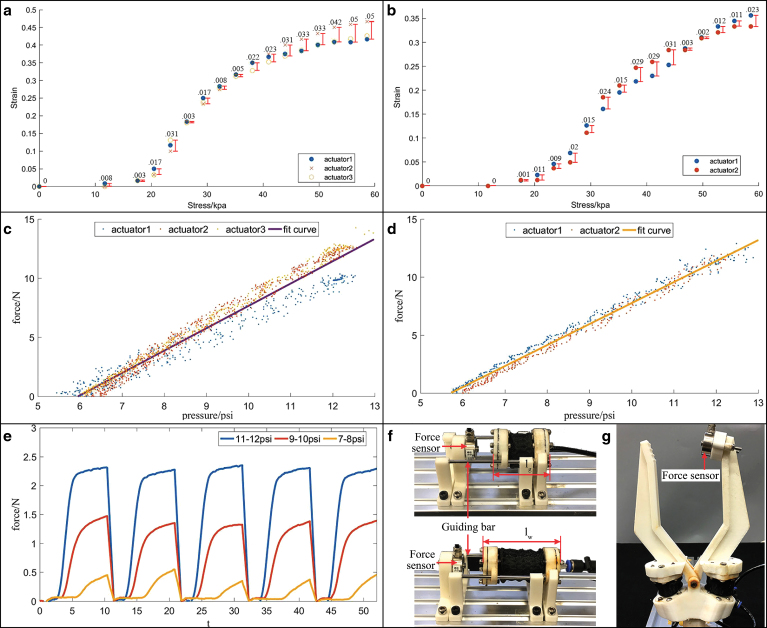
Soft actuator and gripping force test. **(a, b)** Soft actuator stress versus strain test results (wrist and gripper actuators). **(c, d)** Soft actuator force versus pressure test results (wrist and gripper actuators). **(e)** Hybrid gripper gripping force test results (with different inlet CSB pressures). **(f)** Soft actuator validation test setup (*upper*: gripper actuator; *lower*: wrist actuator). **(g)** Gripper test setup. The force sensor was mounted to one finger of the gripper; no further restrictions were added, except for mounting the force sensor to the one finger, the rest of the gripper was in its natural working state. Color images are available online.

As illustrated in the Experimental Setup section, the five actuators used in the manipulator followed the same design for the purpose of modularization and simplicity. To justify the feasibility of this design, the actuators’ stress versus strain, and pressure versus force relationships were tested. The testing setup is shown in Figure 8f. All the parts were fixed except one end of the actuator could move along the guiding bar. The friction between the guiding bar and the actuator was negligible (<0.1 N).

The results for stress versus strain tests are shown in Figure 8a and b. Both of the two kinds of actuators had little elongation at the very beginning. The deformation became more significant when the pressure reached between 30 and 40 kpa for both wrist actuators and gripper actuators. As the pressure kept increasing, the elongation became slow again. Actuators in the same group showed little difference in the stress versus strain relationship. For wrist actuators, the mean error was 0.022, and for gripper actuators, the mean error was 0.0138. Pressure versus force validation results are shown in Figure 8c and d, both fitting curves fitted well with measured data. For the wrist actuators, the RMSE was 0.58, 0.48, and 0.26 N, respectively, and the overall RMSE was 0.81 N. For the gripper actuators, the RMSE was 0.49 and 0.39 N, respectively, and the overall RMSE was 0.44 N.

### Hybrid underwater gripper experiments

A bench test was first carried out to evaluate the gripper's gripping force. A force sensor was mounted at the tip of the gripper (Fig. 8g) and the inlet CSB was set to three different pressure ranges to see the CSBs influence on the gripping force. As shown in Figure 8e, the CSBs working pressure and gripping force had a positive relationship, illustrating that the gripping force could be modified by adjusting the CSBs working pressure ranges.

**FIG. 9.**
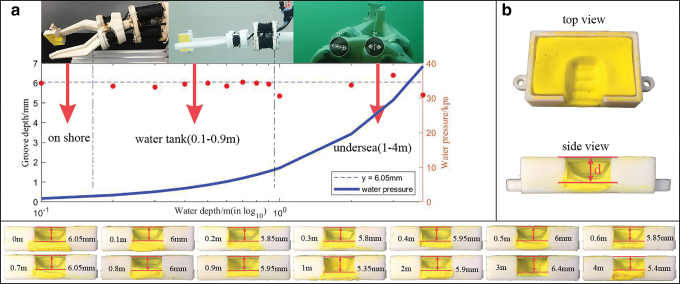
The proposed manipulation system test under different water depths. **(a)** Testing results. The test was carried out onshore, in a water tank, and in the sea. **(b)** Clay box used to demonstrate gripping force. The *bottom* listed clay boxes with all tested depths. Color images are available online.

The manipulation system was also tested under different water depths to demonstrate that the CSB-based hydraulic system was decoupled with water depth. A 3D printed box filled with clay was used for force measurement (Fig. 9b) based on three reasons: (1) Ability to retain its shape after testing and after leaving the water, and for long-term records (in contrast, spring scale will return to normal length once gripping was released). (2) Directly comparable results between different locations and depths, easily visualizable in postanalysis. (3) Being a passive device, no additional power or wiring was required, simple to implement in multisite tests. The box was mounted at the tip of the gripper (Fig. 9a), and the depth of the groove was measured on behalf of the gripping force (Fig. 9b). To limit variables, all boxes were weighted between 10.1 and 10.2 g, the gripping time was set to 15 s, and CSBs working pressure was set to 13–14 psi. This test was carried out in three different places: onshore, in a water tank, and undersea; the onshore test was used as a reference and three boxes were used for each depth.

The results are shown in Figure 9a. The groove depth measured onshore was 6.05 mm (blue dashed line), and the rest points were evenly distributed along the line, with an RMSE of 0.3 mm. The conclusion can be drawn that the CSB-based hydraulic system was independent of ambient water depth.

### Hybrid underwater manipulation system experiments

**FIG. 10.**
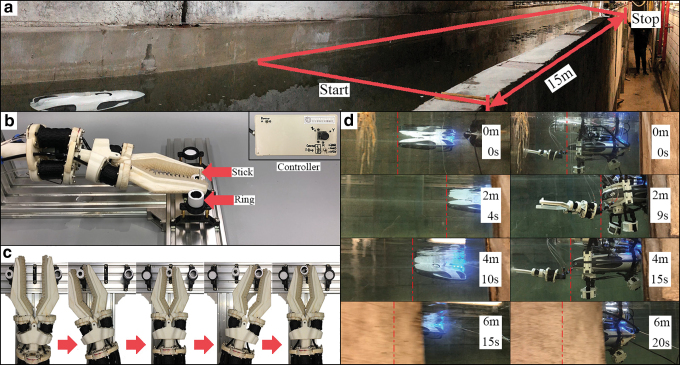
Hybrid manipulator system validation. **(a)** Fifteen-meter water tunnel for speed test. **(b)** Manipulator dexterity and accuracy test (pick and place) setup (onshore). **(c)** Pick and place procedure. **(d)** Comparison between a single ROV and the same ROV with the proposed underwater manipulation system in the speed test. Color images are available online.

The experimental setup is shown in Figure 10b. Two 3D printed rings were placed at two sides and a thin long stick was in the middle. One needed to control the manipulator using the controller and picked up two rings and placed them through the stick in the middle (Fig. 10c). To quantify overall system performance, assuming the inner diameter of the ring was *D*, and the diameter of the stick was 3 mm, if the manipulator could complete the task, the accuracy would be D−3 mm. This task was carried out both onshore and underwater (Supplementary Video S1). In both cases, the manipulator was able to complete the task with an accuracy of 1 mm.

To evaluate the manipulation system's influence on underwater platforms, the whole system was mounted on a consumer-grade ROV for a speed test. The ROV was weighted about 3.8 kg, and the size was 465 × 270 × 126 mm. The ROV was required to travel a 15 m water tunnel as shown in Figure 10a. One round with the manipulation system (Fig. 10d, right) and the other without (Fig. 10d, left). Due to the tensile force on the cable, and the difficulty of direction control in the narrow tunnel when velocity was too high, the ROVs power rating was set to medium. Both setups were tested three times. The results showed that when the manipulation system was on board, the average velocity (0.28 m/s) was 37.8% slower than that when there was only the ROV (0.45 m/s). One main reason for velocity loss was because the manipulation system largely increased the cross-sectional area and destroyed the streamline shape of the ROV. Given the speed of ocean current (0.08–0.25 m/s),^36^ the results demonstrated that mounting the manipulator would lead to a reduced speed of the ROV but still within a viable range.

## Conclusions and Future Work

An underwater hybrid manipulator with a lightweight CSB-based hydraulic actuation control system was proposed in this article. The hybrid manipulator was designed such that the soft actuator design was decoupled with manipulator motion generation. Two CSBs were added into the hydraulic system for the purpose of equalizing ambient water pressure, reducing system bulkiness, and improving dynamic response. An analytical model was elaborated to explore the CSBs performance with respect to deformation, pressure, and flow rate, with well-fitting experimental results. Also, the tracking performance of the proposed hydraulic system was tested, and compared with no CSB condition, the CSBs could significantly improve system performance. Stress versus strain, and pressure versus force relationships for the soft actuator were also tested, and different individuals showed limited differences, justifying the feasibility of this modularized design. Then, the hybrid manipulation system was tested both onshore and underwater (see Appendix) with respect to gripping force and accuracy. The results showed that water depth did not have significant effects on the manipulator's performance, and the proposed underwater manipulation system could handle underwater tasks with good accuracy even without precise control feedback. Finally, a speed test was carried out to evaluate the system's compactness, and it turned out that even a consumer-grade ROV could travel with reasonable speed (0.28 m/s) with the proposed underwater manipulation system.

This project has proven that with the proposed hybrid manipulator and CSB-based hydraulic system, depth is no longer a factor that determines underwater manipulation performance, and even small underwater platforms (<4 kg) can manage complex underwater sampling tasks as good as heavy and expensive setups. The proposed design concepts of the combined manipulator and hydraulic system could serve as a benchmark for reducing system bulkiness and expenses, as large and costly underwater platforms are no longer an essential unit in shallow water sampling. Also, the enabling of small commercial available underwater platforms largely broadens the demographics of this field. The authors believe that the capability of performing underwater sampling with small size devices has great potential in underwater biology study; the low cost also makes it appealing for a much wider audience group.

## Supplementary Material

Supplemental data

## Figures and Tables

**Figure:**
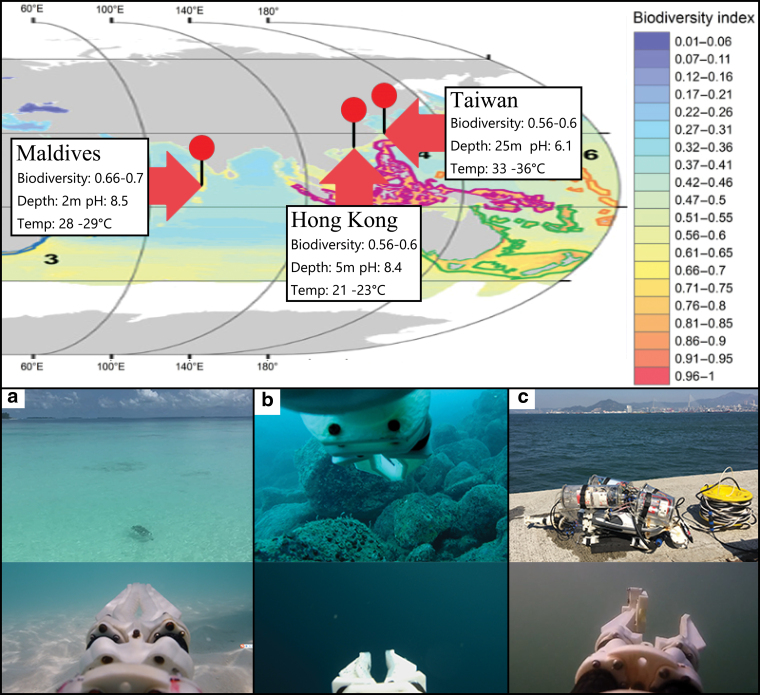
The proposed underwater manipulation system validation studies in three hot spots of marine biodiversity with different hydrographic and climatic conditions. **(a)** Maldives, Kandima Island coral lagoon. **(b)** Taiwan, Guishan Island. **(c)** Hong Kong, west coast. Color images are available online.

## Appendix

The developed prototype of the proposed soft robotic manipulator system has been field tested in three tropical and subtropical locations (Appendix Fig. A1). These places are hot spots of marine biodiversity,^5^ and the hydrographic and climatic conditions are as follows:

(1) Hong Kong, tropical water (pH 8.4) with low temperature (21–23°C) and low salinity (27–28 PSU for open seawater, salinity not applicable for indoor tank freshwater)—four testing locations: (a) indoor water tank with shallow depth and still water, (b) indoor water tunnel, (c) south beach, and (d) western pier dockland.
(2) Taiwan, Guishan Island, subtropical water (25 m), active undersea volcano harsh environment [pH = 6.1, strong acid (sulfur)], highest temperature (33–36°C).
(3) Maldives, Kandima Island coral lagoon, shallow (2 m) equatorial water (pH 8.5) with medium temperature (28–29°C) and high salinity (34 PSU).

## References

[1] Hamner W, Madin L, Alldredge A, *et al.* Underwater observations of gelatinous zooplankton: sampling problems, feeding biology, and behavior. Limnol Oceanogr 1975;20:907–917

[2] Xu Y, Richlen ML, Morton SL, *et al.* Distribution, abundance and diversity of gambierdiscus spp. from a ciguatera-endemic area in Marakei, Republic of Kiribati. Harmful Algae 2014;34:56–68

[3] Parham D Scientific Diving: Code of Conduct. Poole, United Kingdom: Centre for Marine and Coastal Archaeology, School of Conservation Sciences, Bournemouth University, 2006

[4] Pardo A A scuba diving direct sediment sampling methodology on benthic transects in glacial lakes: procedure description, safety measures, and tests results. Environ Sci Pollut Res 2014;21:12457–12471 10.1007/s11356-014-3011-8 24943883

[5] Ramírez F, Afán I, Davis LS, *et al.* Climate impacts on global hot spots of marine biodiversity. Sci Adv 2017;3:e1601198 28261659 10.1126/sciadv.1601198 PMC5321448

[6] Khatib O, Yeh X, Brantner G, *et al.* Ocean one: a robotic avatar for oceanic discovery. IEEE Robot Autom Mag 2016;23:20–29

[7] Stuart H, Wang S, Khatib O, *et al.* The ocean one hands: an adaptive design for robust marine manipulation. Int J Robot Res 2017;36:150–166

[8] Yuh J Design and control of autonomous underwater robots: a survey. Auton Robots 2000;8:7–24

[9] Fernández JJ, Prats M, Sanz PJ, *et al.* Developing a new underwater robot arm for shallow-water intervention. IEEE Robot Autom Mag 2013;1070:121–130

[10] Marani G, Choi SK, Yuh J Underwater autonomous manipulation for intervention missions auvs. Ocean Eng 2009;36:15–23

[11] Rus D, Tolley MT Design, fabrication and control of soft robots. Nature 2015;521:467 26017446 10.1038/nature14543

[12] Laschi C, Mazzolai B, Cianchetti M Soft robotics: technologies and systems pushing the boundaries of robot abilities. Sci Robot 2016;1:eaah3690 10.1126/scirobotics.aah3690 33157856

[13] Wang Y, Yang X, Chen Y, *et al.* A biorobotic adhesive disc for underwater hitchhiking inspired by the remora suckerfish. Sci Robotics 2017;2:eaan8072 10.1126/scirobotics.aan8072 33157888

[14] Shen Z, Na J, Wang Z A biomimetic underwater soft robot inspired by cephalopod mollusc. IEEE Robot Autom Lett 2017;2:2217–2223

[15] Shen Z, Yi J, Li X, *et al.* A soft stretchable bending sensor and data glove applications. Robot Biomim 2016;3:22 10.1186/s40638-016-0051-1 PMC5133288 28003951

[16] Shintake J, Cacucciolo V, Floreano D, *et al.* Soft robotic grippers. Adv Mater 2018;1707035 10.1002/adma.201707035 29736928

[17] Lane DM, Davies JBC, Casalino G, *et al.* Amadeus: advanced manipulation for deep underwater sampling. IEEE Robot Autom Mag 1997;4:34–45

[18] Lane DM, Davies JBC, Robinson G, *et al.* The amadeus dextrous subsea hand: design, modeling, and sensor processing. IEEE J Ocean Eng 1999;24:96–111

[19] Stuart HS, Wang S, Gardineer B, et al. A Compliant Underactuated Hand with Suction Flow for Underwater Mobile Manipulation (2014 IEEE International Conference on Robotics and Automation—ICRA). Hong Kong: IEEE, 2014, pp. 6691–6697

[20] Calisti M, Giorelli M, Levy G, *et al.* An octopus-bioinspired solution to movement and manipulation for soft robots. Bioinspir Biomim 2011;6:036002 21670493 10.1088/1748-3182/6/3/036002

[21] Cianchetti M, Arienti A, Follador M, *et al.* Design concept and validation of a robotic arm inspired by the octopus. Mater Sci Eng C 2011;31:1230–1239

[22] Cianchetti M, Calisti M, Margheri L, *et al.* Bioinspired locomotion and grasping in water: the soft eight-arm octopus robot. Bioinspir Biomim 2015;10:035003 25970014 10.1088/1748-3190/10/3/035003

[23] Arienti A, Calisti M, Giorgio-Serchi F, *et al.* Poseidrone: Design of a Soft-Bodied Rov with Crawling, Swimming and Manipulation Ability. In: OCEANS - San Diego. (Oceans) San Diego: IEEE, 2013, pp. 1–7

[24] Giannaccini ME, Xiang C, Atyabi A, *et al.* Novel design of a soft lightweight pneumatic continuum robot arm with decoupled variable stiffness and positioning. Soft Robot 2018;5:54–70 29412080 10.1089/soro.2016.0066 PMC5804101

[25] Stilli A, Wurdemann HA, Althoefer K Shrinkable, Stiffness-Controllable Soft Manipulator Based on a Bio-Inspired Antagonistic Actuation Principle (2014 IEEE/RSJ International Conference on Intelligent Robots and Systems—IROS 2014). Chicago: IEEE, 2014, pp. 2476–2481

[26] Kurumaya S, Phillips BT, Becker KP, *et al.* A modular soft robotic wrist for underwater manipulation. Soft Robot 2018;5:399–409 29672216 10.1089/soro.2017.0097

[27] Teoh ZE, Phillips BT, Becker KP, *et al.* Rotary-actuated folding polyhedrons for midwater investigation of delicate marine organisms. Sci Robot 2018;3:eaat5276 10.1126/scirobotics.aat5276 33141728

[28] Galloway KC, Becker KP, Phillips B, *et al.* Soft robotic grippers for biological sampling on deep reefs. Soft Robot 2016;3:23–33 27625917 10.1089/soro.2015.0019 PMC4997628

[29] Vogt DM, Becker KP, Phillips BT, *et al.* Shipboard design and fabrication of custom 3d-printed soft robotic manipulators for the investigation of delicate deep-sea organisms. PLoS One 2018;13:e0200386 30067780 10.1371/journal.pone.0200386 PMC6070194

[30] Suzumori K, Wada A, Wakimoto S New mobile pressure control system for pneumatic actuators, using reversible chemical reactions of water. Sens Actuators A Phys 2013;201:148–153

[31] Licht S, Collins E, Ballat-Durand D, *et al.* Universal Jamming Grippers for Deep-Sea Manipulation (OCEANS 2016 MTS/IEEE Monterey) IEEE, 2016, pp. 1–5

[32] Licht S, Collins E, Mendes ML, *et al.* Stronger at depth: jamming grippers as deep sea sampling tools. Soft Robot 2017;4:305–316 29251570 10.1089/soro.2017.0028

[33] Phillips BT, Becker KP, Kurumaya S, *et al.* A dexterous, glove-based teleoperable low-power soft robotic arm for delicate deep-sea biological exploration. Sci Rep 2018;8:14779 30283051 10.1038/s41598-018-33138-y PMC6170437

[34] Gaiser I, Wiegand R, Ivlev O, *et al.* Compliant Robotics and Automation with Flexible Fluidic Actuators and Inflatable Structures (Smart Actuation and Sensing Systems-Recent Advances and Future Challenges). In: Berselli G, Vertechy R, Vassura G (Eds). London: IntechOpen, 2012

[35] Polygerinos P, Wang Z, Overvelde JT, *et al.* Modeling of soft fiber-reinforced bending actuators. IEEE Trans Robot 2015;31:778–789

[36] Greene C Oceanography: a view of the earth. IEEE J Ocean Eng 1987;12:535–535

